# Rural to urban migration is associated with increased prevalence of childhood wheeze in a Latin-American city

**DOI:** 10.1136/bmjresp-2017-000205

**Published:** 2017-07-03

**Authors:** Alejandro Rodriguez, Maritza G Vaca, Martha E Chico, Laura C Rodrigues, Mauricio L Barreto, Philip J Cooper

**Affiliations:** 1 Laboratorio de Investigación FEPIS, Quinindé, Ecuador; 2 Faculty of Epidemiology and Population Health, London School of Hygiene and Tropical Medicine, London, UK; 3 Centro de Pesquisas Gonçalo Muniz, FIOCRUZ, Salvador, Brazil; 4 Instituto de Saude Coletiva, Universidade Federal da Bahia, Salvador, Brazil; 5 Facultad de Ciencias Medicas, de la Salud y la Vida, Universidad Internacional del Ecuador, Quito, Ecuador; 6 Institute of Infection and Immunity, St George’s University of London, London, UK

**Keywords:** wheeze, asthma, internal migration, Latin America

## Abstract

**Introduction:**

The urbanisation process has been associated with increases in asthma prevalence in urban and rural areas of low-income and middle-income countries (LMICs). However, although rural to urban migration and migration between cities are considered important determinants of this process, few studies have evaluated the effects of internal migration on asthma in urban populations of LMICs. The present study evaluated the effects of internal migration on the prevalence of wheeze in an urban area of Latin America.

**Methods:**

We did a cross-sectional analysis of 2510 schoolchildren living in the city of Esmeraldas, Ecuador. Logistic regression was used to analyse associations between childhood wheeze and different aspects of migration among schoolchildren.

**Results:**

31% of schoolchildren were migrants. Rural to urban migrants had a higher prevalence of wheeze, (adj.OR=2.01,95% CI1.30 to 3.01, p=0.001) compared with non-migrants. Age of migration and time since migration were associated with wheeze only for rural to urban migrants but not for urban to urban migrants. Children who had migrated after 3 years of age had a greater risk of wheeze (OR 2.51, 95% CI 1.56 to 3.97, p=0.001) than non-migrants while migrants with less than 5 years living in the new residence had a higher prevalence of wheeze than non-migrants (<3 years: OR=2.34, 95% CI 1.26 to 4.33, p<0.007 and 3–5 years: OR=3.03, 95% CI 1.49 to 6.15, p<0.002).

**Conclusions:**

Our study provides evidence that rural to urban migration is associated with an increase in the prevalence of wheeze among schoolchildren living in a Latin-American city. Age of migration and time since migration were important determinants of wheeze only among migrants from rural areas. A better understanding of the social and environmental effects of internal migration could improve our understanding of the causes of the increase in asthma and differences in prevalence between urban and rural populations.

**key messages**

- Internal migration process could be related with the increase and differences in asthma prevalence between urban and rural areas of Latin-America.
- Few studies have evaluated the effect of internal migration on asthma in an urban area of low-income and middle-income countries.
- Our findings show how some migrant categories based on temporal and spatial characteristics area associated with asthma/wheeze prevalence.

## Introduction

Over the past 40 years or so, there has been a progressive increase in the prevalence of asthma and other allergic diseases particularly in high-income countries (HICs) and in urban areas of HICs and low-income and middle-income countries (LMICs).^1^ However, in recent years the prevalence of allergic disorders may have reached a plateau in some HICs but continues to increase in LMICs.^2^ The reasons for these variations remain unexplained but are likely to be caused by a complex interplay of biological, environmental and social factors.^3 4^

Urbanisation is a social process that has been causally implicated in trends of increasing asthma prevalence in LMICs,^3–6^ and the lower prevalence in rural compared with urban populations.^3 7^ Rural to urban differences in asthma prevalence have been attributed to the protective effects of environmental exposures such as farming that are typical of a rural way of life.^8^ However, recent studies have shown that allergic disorders may be increasing in rural areas thus reducing the prevalence gap between urban and rural settings.^9 10^ Furthermore, an array of environmental and social changes stemming from the urbanisation process have been identified as potential risk factors for asthma in urban and rural areas.^4 8^

Rural to urban migration and migration between cities are important components of the urbanisation process in LMICs and are related to environmental, socioeconomic and behavioural changes.^11^ However, few epidemiological studies have investigated the influence of internal migration on asthma and other allergic diseases in LMICs.^12 13^ Most studies of the effects of migration on allergic diseases have investigated populations migrating from LMICs (presumed low risk for allergic diseases) to HICs (presumed high risk).^14 15^ These studies have shown that being born in an area of low risk provides protection against asthma,^16 17^ but this protection may decline with the length of residence in the new environment.^18^ Others studies have shown that age of migration and time since migration are associated with the risk of asthma and other allergic diseases,^14^ often leading to a higher risk of atopy and allergic diseases among migrants than the local population.

Although internal migration can be defined as the movement of an individual between two geographical locations (rural to urban or urban to urban) of the same country or region,^19^ the social, economic and environmental conditions of migrant populations transform this simple movement into a more complex process with different effects on health.^20^ Temporal, spatial and social characteristics of the population produce different groups of migrants each with specific features with potentially differing effects on asthma risk. Further, the consequences of migration are relevant to individual migrants and to their families and communities both in place of origin and destination.^21^ A better understanding how these factors, relating to the migration process may alter risk of asthma and other allergic diseases, may contribute to our  comprehension of the causes of temporal increases in asthma prevalence and the differences in prevalence between urban and rural populations of Latin America (LA).^22 23^ The aim of the present study was to explore the effects of internal migration on the prevalence of wheeze in schoolchildren living in a coastal city in Ecuador.

## Methods

### Study population

The study was conducted in the city of Esmeraldas, the provincial capital of the tropical coastal province of Esmeraldas in north-western Ecuador, located 140 km south of the Colombian border. With approximately 190 000 inhabitants, Esmeraldas is the principal northern port and is home to the country’s largest oil refinery. The main economic and industrial activities of the population are based on oil processing and export, commerce, agriculture (especially tropical fruit and palm oil), timber, fishing and tourism. Based on the last national census of 2010, the coverage of basic services in the city is deficient: 28% of the households have no access to running water, 22% lack a sewage system and 5% have no access to electricity. The educational level of the population is low compared with the national average: 6% of the population is illiterate while only 18% has higher education.^24^

### Study design and sample

A cross-sectional study was conducted in schoolchildren aged 5–16 years to evaluate risk factors for allergy and asthma and history of migration.^12^ A convenience sample of 10 schools was selected from nine barrios or neighbourhoods in which there was a predominance of Afro-Ecuadorian migrants from the rural districts of the province. . All children attending the schools at the time of the survey were eligible for inclusion. The response rate was 90.8% (of the annually updated school lists). Data collection was done between November 2007 and January 2010.

### Data collection

Detailed information on risk factors and symptoms of wheeze was collected using a questionnaire based on the International Study of Asthma and Allergies in Childhood (ISAAC) phase II.^25^ The questionnaire, translated into Spanish and adapted to local conditions, was administered to the parent or guardian of each child by trained field workers. Wheeze was defined as a positive response to the question ‘Has your child had wheeze in the chest in the last 12 months’. The questionnaire was also used to collect information on migration history of the children and their parents.

### Migrant categories

Migration data were analysed based on the recommendations of the United Nations Secretariat to measure internal migration.^19^ Detailed information about place of birth (community/city, parish, province and country) and temporal characteristics of migratory movements were collected for each child. Migration was defined as a change of residence from one civil division to another in which community was the minor division. *Migration status* was measured based on the place of birth of the children by the question ‘*Where was the child born?*’ Children who had been born in the city of Esmeraldas were treated as non-migrants and all others as migrants. Migrants were classified into several categories based on spatial and temporal characteristics of their migratory movements: (1) *Direction of migration* classified migrants by direction of migration between place of birth and current place of residence (rural to urban or urban to urban), (2) *Locality of migration* classified migrants by the population size at the place of birth. (Categories explained in table 1), (3). *Age of migration* classified migrants by age when they left their place of birth (≤3 years and >3 years), and (4) *Time since migration* classified migrants by time spent in the city of Esmeraldas (<3 years, 3–5 years and >5 years). Migrant status of parents was also included. Categories and definitions are provided in table 1. Parents were treated as non-migrants if they were born in the city of Esmeraldas and as migrants if they were born elsewhere. Due to the proximity of the city of Esmeraldas to the international border with Colombia (140 km), the variable ‘Colombian children’ was included also representing children with a history of migration from Colombia as first-generation or second-generation international migrants (ie, children born in Colombia or born to migrant Colombian parents). Additional data collected for each child included age, sex, parents living in the child’s home (both, one, none), farm environment (households characterised by farming activities and presence of peridomestic animals), type of house (*urban house*: residences with connection to running water, concrete building materials for walls, presence of a flushing toilet, ownership of a set of electric appliances and two to three urban services; *transitional house*: residences with an incomplete set of electric appliances, latrine for bathroom, use of mixed materials for house construction) and consumption of junk food (consumption of fizzy drinks). Variables to represent farm environment and type of housing were created using multiple correspondence analysis (MCA) and methodology is explained elsewhere.^26^ The variables used in MCA to define ‘farm environment’ were parental agricultural activities, contact with animals in farms and animal breeding in or around the household. Variables to define ‘type of house’ were basic services, disposal of faeces, electrical appliances, household construction materials and type of cooking fuel (table 1).

**Table 1:** History of migration, demographic variables and socioeconomic variables

Dimensions/ indicators	Definitions	Categories	n	Total population	Migrant population
Migrant status	Classified children by difference between place of birth and place of residence	NMa	1694	68.7%	
		Migrant	772	31.3%	100%
Direction of migration	Classified migrants by migration movement between place of birth and place of residence based on the political division of Ecuador	NM	1694	68.7%	
		Rural to urban	286	11.6%	37%
		Urban to urban	486	19.7%	63%
Locality of migration	Classified migrants by size of population of place of birth: *communities* (rural village), *small city* (urban towns*), medium city* (provincial capitals) and *large city* (migrants from Quito and Guayaquil, the two largest cities in the country)	NM	1694	68.7%	
		Community	286	11.6%	37%
		Small city	170	6.9%	22%
		Medium city	124	5%	16%
		Large city	192	7.8%	25%
Age at migration	Classified migrants considering the age when children left their place of birth (years)	NM	1694	68.7%	
		≤3	291	11.8%	38%
		>3	481	19.5%	62%
Time since migration	Classified migrants by time spent in current locality (years)	NM	1694	68.7%	
		<3	275	11.2%	36%
		3–5	178	7.2%	23%
		>5	319	12.9%	41%
Migration status of parents	Parents were treated as non-migrants if they were born in the city of Esmeraldas and as migrants if they were born elsewhere	NM	575	24%	
		One migrant parent	877	36%	
		Two migrant parents	981	40%	
Colombian children	First-generartion or secondgeneration migrants from Colombia	No	2332	92.9%	
		Yes	103	4.1%	
Age of the children	Age of the children (years)	≤9	1294	51.6%	
		>10	1216	48.4%	
Sex	Sex of the child	Male	1321	52.7%	
		Female	1189	47.4%	
Farm environment	Classified houses of the children based on farm characteristics (eg, presence of animals and agricultural activities)^26^	No	2141	85.3%	
		Yes*	369	14.7%	
Type of house	Classified houses of the children based on construction materials, presence of urban services and electrical appliances^26^	Urban†	2190	87.3%	
		Transitional‡	320	12.7%	
Consumption of junk food	Classified children by fizzy drink consumption	Barely	738	33.7%	
		Sometimes/week	1132	51.6%	
		Daily	323	14.7%	
Parents living in the child’s house	Classified children based on the presence of parents at home	Both	1138	45%	
		One	889	36%	
		None	483	19%	

*Houses characterised by farm activities and peridomestic animal breeding.

†Urban house: residences with connection to running water, concrete building materials for walls, use of a flushing toilet, ownership of a set of appliances and two to three urban services.

‡Transitional: residences with an incomplete set of electric appliances, latrine bathroom and use of mixed materials in house construction.

a NM: no migrant

### Statistical analysis

Logistic regression was used to explore associations between migrant categories and wheeze in the last 12 months. Each migrant category was adjusted for age, sex, farm environment, type of house and consumption of junk food. Associations with p<0.05 were considered statistically significant. All analyses were done using SPSS V.23.

## Results

We evaluated a total of 2510 schoolchildren in the city of Esmeraldas of whom 44 were excluded due to lack of information on child’s birthplace. The prevalence of wheeze in the last 12 months was 9.4%.

### History of migration

Almost a third (31.3%) of schoolchildren were migrants (table 1). Among migrants, 63% had a history of urban to urban migration and 37% of rural to urban migration, while 25% came from large cities (Quito or Guayaquil), 16% from medium cities (provincial capitals), 22% from small cities (city towns) and 37% from rural communities. Age at migration was most frequently reported after 3 years of age (62%) and most migrants (41%) had lived more than 5 years in the city of Esmeraldas. Of the total study population, 4% were first-generation or second-generation international migrants from Colombia, and history of parental migration was reported for 76%.

### Sociodemographic characteristics of the migrant population

A higher proportion of migrants than non-migrants was female (45.1 vs 52.6%) while rural to urban migrants tended to be older than the other groups (table 2). Farm environment was more common in migrants (17.3%) than non-migrants (13.6%). Only 25% of the rural to urban migrant children were living with both parents compared with 52% for non-migrant children. Daily consumption of junk food was greater in non-migrants.

**Table 2:** Demographic and socioeconomic variables by direction of migration

Variables	Categories	Direction of migration of children						
		Non-migrant		Rural to urban		Urban to urban		χ^2^
		n	%	n	%	n	%	p Value
Migration status of parents	NM	466	28.4%	20	7.2%	75	15.9%	<0.001
	One migrant parent	600	36.6%	83	30.1%	182	38.5%	
	Two migrant parents	574	35.0%	173	62.7%	216	45.7%	
Colombian children	No	1598	97.4%	270	97.8%	420	88.4%	<0.001
	Yes	42	2.6%	6	2.2%	55	11.6%	
Age of children (years)	≤9	901	53.2%	121	42.3%	246	50.6%	0.003
	>10	793	46.8%	165	57.7%	240	49.4%	
Sex of children	Male	930	54.9%	130	45.5%	239	49.2%	0.003
	Female	764	45.1%	156	54.5%	247	50.8%	
Farm environment	Non-farm	1464	86.4%	236	82.5%	403	82.9%	0.059
	Farm	230	13.6%	50	17.5%	83	17.1%	
Type of house	Basic urban	1491	88.0%	242	84.6%	416	85.6%	0.148
	Transitional	203	12.0%	44	15.4%	70	14.4%	
Consumption of junk food	Barely	525	35.3%	73	31.7%	129	29.9%	0.041
	Sometimes/week	733	49.3%	130	56.5%	243	56.4%	
	Daily	230	15.5%	27	11.7%	59	13.7%	
Parents living in child’s house	Both	876	51.7%	70	24.5%	176	36.2%	<0.001
	One	580	34.2%	103	36.0%	183	37.7%	
	None	238	14.0%	113	39.5%	127	26.1%	

### Associations between history of migration and wheeze

Positive associations were observed with direction of migration and locality of migration in unadjusted analyses (table 3). Rural to urban migrant children (or migrants from communities) had greater odds of wheeze than non-migrants (OR 1.66, 95% CI 1.15 to 2.41, p=0.007). Children with a history of migration from Colombia had a higher prevalence of wheeze (OR 1.83, 95% CI 1.05 to 3.18, p=0.032) compared with Ecuadorian children. Multivariable analyses were adjusted for sex, age, farm environment, type of housing and consumption of junk food. Adjusted analyses showed a higher risk of wheeze compared with non-migrant children for the following: (1) Direction and locality of migration—children who migrated from rural (or communities) to urban areas (OR 2.01, 95% CI 1.30 to 3.01, p=0.001) had two times more wheeze than non-migrant children; (2) History of migration of the parents—children for whom both parents had a history of migration had a greater risk of wheeze than children whose parents had not (OR 1.52, 95% CI 1.01 to 2.29, p=0.046) and (3) Colombian children had greater risk of wheeze (OR 2.15, 95% CI 1.22 to 3.78, p=0.008) than Ecuadorian children.

**Table 3:** ORs and 95% CIs for associations between wheeze in the last 12 months and history of migration adjusted for age, sex and socioeconomic variables

		Wheeze	Univariable			Multivariable		
Dimensions	Categories	Prevalence	OR	(95% CI)	p	OR*	(95% CI)	p
Migration status	Non-migrant	9.1%	1			1		
	Migrant	10.4%	1.15	(0.86 to 1.53)	0.342	1.25	(0.91 to 1.71)	0.175
Direction of migration	Non-migrant	9.1%	1			1		
	Rural to urban	14.3%	1.66	(1.15 to 2.41)	0.007	2.01	(1.30 to 3.01)	0.001
	Urban to urban	8%	0.87	(0.60 to 1.25)	0.443	0.91	(0.61 to 1.63)	0.660
Locality of migration	Non-migrant	9.1%	1			1		
	Communities	14.3%	1.66	(1.15 to 2.41)	0.007	2.01	(1.30 to 3.01)	0.001
	Small city	5.3%	0.56	(0.28 to 1.11)	0.095	0.59	(0.29 to 1.20)	0.145
	Medium city	12.1%	1.37	(0.78 to 2.40)	0.278	1.68	(0.92 to 3.07)	0.089
	Large city	7.8%	0.84	(0.48 to 1.46)	0.540	0.80	(0.42 to 1.52)	0.486
Age at migration	Non-migrant	9.1%	1			1		
	≤3 vs NM	10.3%	1.14	(0.75 to 1.73)	0.530	1.20	(0.76 to 1.89)	0.437
	>3 vs NM	10.4%	1.15	(0.82 to 1.61)	0.410	1.28	(0.86 to 1.87)	0.204
Time since migration (years)	Non-migrant	9.1%	1			1		
	<3	12.4%	1.40	(0.94 to 2.08)	0.095	1.36	(0.87 to 2.13)	0.185
	3–5	9%	0.98	(0.57 to 1.68)	0.943	1.12	(0.63 to 1.98)	0.705
	>5	9.4%	1.03	(0.68 to 1.55)	0.885	1.23	(0.77 to 1.94)	0.388
History of migration of the parents	Non-migrant	7.8%	1			1		
	One migrant parent	9.5%	1.23	(0.84 to 1.80)	0.282	1.39	(0.92 to 2.11)	0.123
	Two migrant parents	10.3%	1.35	(0.94 to 1.95)	0.108	1.52	(1.01 to 2.29)	0.046
Colombian children	No	9.1%	1			1		
	Yes	15.5%	1.83	(1.05 to 3.18)	0.032	2.15	(1.22 to 3.78)	0.008

*OR adjusted by sex, age, farm environment, quality of the house and consumption of junk food.

Table 4 shows the adjusted associations between wheeze and age at migration and time since migration, stratified by direction of migration. Positive and statistically significant associations were observed for rural to urban migrants but not for urban to urban migrants. Among rural to urban migrants, those who had migrated after 3 years of age had a greater risk of wheeze (OR 2.5, 95% CI 1.56 to 3.67, p<0.001) than non-migrant children and those who had spent less than 3 years and between 3–5 years in the new area of residence had a higher prevalence of wheeze than non-migrant children (OR 2.34, 95% CI 1.26 to 4.33, p<0.007 and OR 3.03, 95% CI 1.49 to 6.15, p=0.002, respectively).

**Table 4:** ORs and 95% CIs for associations between wheeze in the last 12 months and age at migration and time since migration, stratified by direction of migration. ORs adjusted for age, sex and socioeconomic variables

		Rural to urban			Urban to urban		
Dimensions	Categories	OR*	(95% CI)	p	OR*	(95% CI)	p
Age at migration (years)	Non-migrant	1			1		
	≤3 vs NM	1.05	(0.44 to 2.49)	0.909	1.30	(0.76 to 2.19)	0.319
	>3 vs NM	2.51	(1.56 to 3.97)	<0.001	0.67	(0.38 to 1.18)	0.164
Time since migration (years)	Non-migrant	1			1		
	<3	2.34	(1.26 to 4.33)	0.007	0.94	(0.51 to 1.73)	0.840
	3–5	3.03	(1.49 to 6.15)	0.002	0.41	(0.15 to 1.13)	0.086
	>5	1.15	(0.54 to 2.46)	0.714	1.28	(0.74 to 2.20)	0.374

*OR adjusted by sex, age, farm environment, quality of the house and consumption junk food.

## Discussion

In the present analysis, we have explored how internal migration affects the prevalence of recent wheeze among schoolchildren living in a city in a coastal tropical area of LA. Clearly, the study of migration is complex because of the spatial and temporal dimensions of migratory movements and also due to different social backgrounds within migrant populations. These characteristics produce several types of migrants each with specific population features.^20 21^ Additionally, migration is a multistage process with effects not only at the individual level, but also at family and community levels.^21^ Considering this, we have taken a multidimensional approach to analyse migration, using categories based on temporary and spatial characteristics of the migratory movements of children and their parents. Our data provide evidence that internal migration, specifically rural to urban migration, was associated with a higher risk of wheeze. Further, international history of migration of the children, specifically migrants from Colombia, was associated with a greater prevalence of wheeze: Colombian children had a twofold greater risk of wheeze compared with Ecuadorian children. Our data extend our previous observations on the effects of migration on allergic diseases in Afro-Ecuadorian schoolchildren living in rural communities in Esmeraldas Province in Ecuador^13^ in which we have shown that migration before 1 year of age and international migration (from nearby areas of the border between Ecuador and Colombia) to a rural community were associated with a higher prevalence of recent wheeze and rhinitis in transitional rural communities. We also observed that the absence of the mother at home, due to temporary or permanent migration, was associated with an increase in the occurrence of wheeze, rhinitis and eczema in rural areas.^13^

Although internal migrants account for nearly four times as many individuals as international migrants,^21^ associations between migration status and asthma and other allergic diseases have generally been investigated in populations migrating between countries, mostly by comparing those that have migrated from LMICs to HICs. Two publications have reviewed studies of differences in prevalence of asthma and other allergic diseases between international migrants, the host population and the population of origin. The first, by Rottem and colleagues who reviewed available literature published before 2003,^14^ concluded from 14 published studies that international migrants from LMICs to HICs tended to develop more allergies and asthma compared with their populations of origin in a time-dependent fashion, had a greater risk if migration occurred before 2 years of age and were more prone to allergies than the host populations. The second (a systematic review by Cabieses and colleagues),^15^ evaluated 54 studies of which 41 were published in the last 10 years. The authors concluded that the prevalence of asthma but not ‘allergies’ was lower in migrants compared with the host population and that the prevalence of asthma tended to converge with that of the host population over time. Further, the study also concluded that asthma prevalence was generally higher in the second-generation compared with the first-generation of migrants. Although the overall conclusions of these reviews of published studies were consistent with the premise that migrants from LMICs suffer less asthma symptoms than host populations for a period following migration, not all studies supported such a conclusion. A recent analysis of data from the ISAAC phase III studies that included study centres from both LMICs and HICs indicated that being born outside the country of residence was associated with a lower prevalence of asthma, rhinoconjunctivitis and eczema but only for migrants to affluent countries.^27^ The results for non-affluent countries showed a higher prevalence of eczema symptoms in migrants and no associations for asthma and rhinoconjunctivitis.^27^

**Figure 1:**
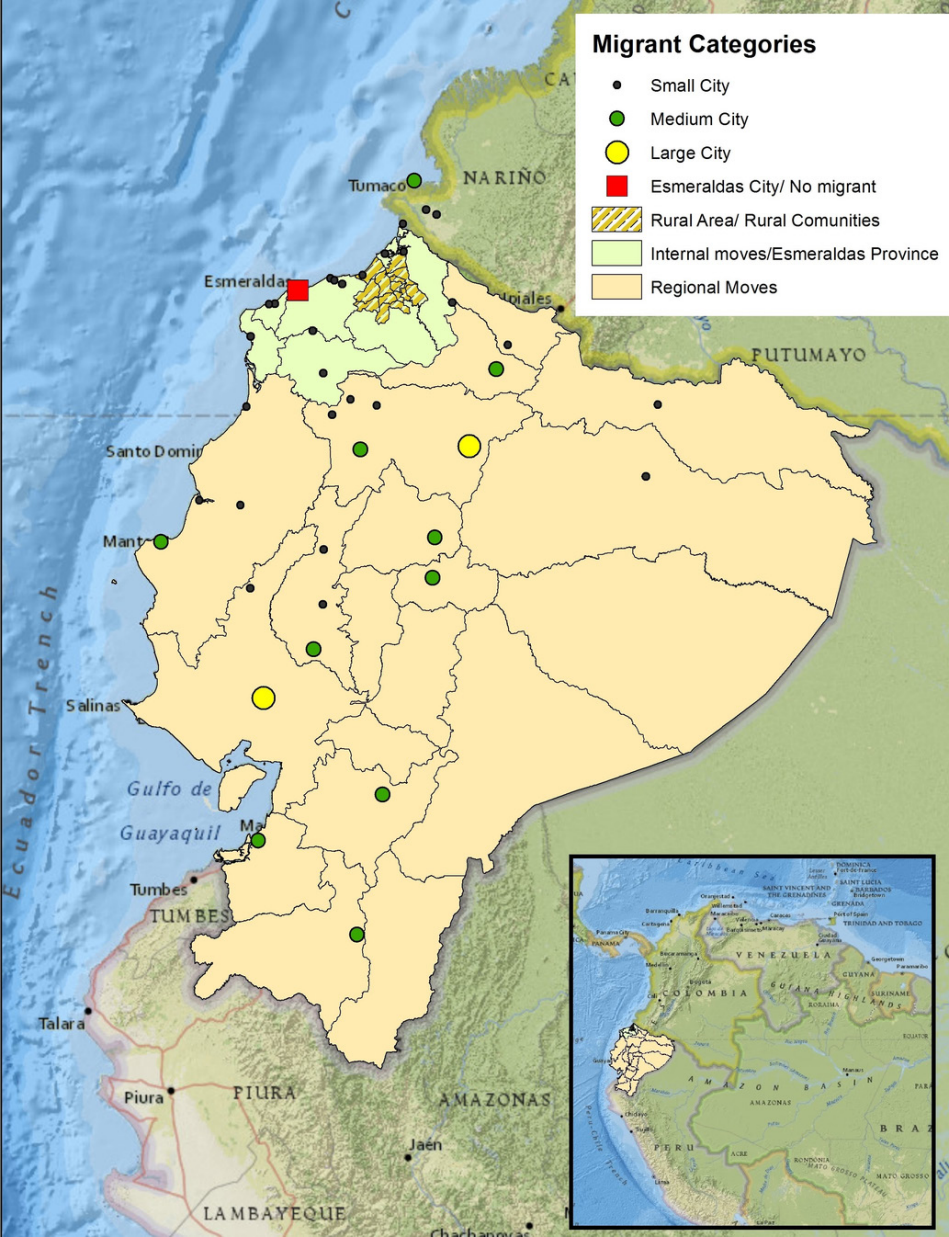
Study site. Map of Ecuador showing migrant categories by location. Red square represents Esmeraldas city (study area), green area represents internal migrations in the Esmeraldas Province and yellow area represents internal moves (migrants of other provinces of Ecuador). Yellow, green and black circles represent large, medium and small cities, respectively.

Studies conducted in Asia and LA have used history of rural residence to evaluate the effects of rural/farm environment on allergic diseases in urban populations.^28 29^ Although these studies did not focus on the study of migration as a risk factor, they provided a good starting point to evaluate the effects of internal migration on asthma in LMICs. The first study, conducted in Mongolia, showed that subjects aged 10–60 years who relocated from a small rural village into a town, were more likely to develop asthma than subjects who lived in a town from birth, although this trend was not statistically significant.^28^ Another study conducted in an urban area of Argentina showed that adolescents aged 13–14 years with a history of rural residence had the same prevalence of wheeze compared with those who had always lived in the urban area.^29^ Our study focused on the effects of internal migration on wheeze prevalence in an urban population of an LMIC, where migrants formed a diverse group including migrants from rural communities, migrants from other urban settings, and those crossing voluntarily or being displaced by civil conflict across the international border with Colombia (figure 1). In this setting, we found that rural to urban migration is an important determinant of a higher risk of wheeze in an urban population. Further, family history of migration was associated with an increase in wheeze prevalence that was especially marked among Colombian children. However, in contrast to previous international comparisons, our data showed that migrants from populations considered to be at low risk for allergic diseases (rural communities) actually had a slightly higher prevalence of wheeze than the host population in the urban area (14.3% vs 9.1%), challenging the assumption that rural residence protects against allergic diseases.^4^ At the same time, the prevalence of asthma in rural migrants was slightly higher (14.3% vs 10.1%) than that of the population of origin (rural communities located north of the city of Esmeraldas),^26^ indicating an increase in risk related to the migration process itself. Another important finding was the effect of time since migration and age of migration in rural migrants: the risk of wheeze increased with greater time since arriving in the city up to 5 years, after which the risk disappeared (table 4). Our data also indicate that age of migration was associated an increased risk of wheeze among children who migrated after 3 years of age.

In an international context, the apparently lower prevalence of asthma and allergic diseases in migrants could be explained by the ‘*healthy migrant effect’* in which recent migrants, including those migrating from LMICs to HICs, are on average healthier than the native population.^30^ International migrants are not a random sample of their country of origin but a highly selected group who are able or motivated to deal with the stress, cost and organisation that such a process entails. Individuals or families who migrate are in a relatively advantageous position, whether financial or social. However, migrant health may deteriorate with increasing length of residence in the new country.^29^ In the case of rural to urban migration, evidence for a healthy migrant effect is limited.^31^ In our study, socioeconomic variables of the migrant population were not statistically different than those of the non-migrant population. However, the tough social conditions that new rural migrants face in LMIC cities could explain partly the higher prevalence of wheeze/asthma in rural migrants. It is well known that rural migrants move to the cities in search of work or to improve their quality of life. Most rural migrants settle at the periphery of growing cities in areas that lack basic services and infrastructure. Such newly established neighbourhoods are characterised by low quality of life, poor housing and poverty.^32^ Several studies conducted in urban centres of LA have shown an increased risk of wheeze/asthma to be associated with factors indicative of poverty, dirt and infections.^33 34^ Thus, the adverse urban environment in which new rural migrants find themselves could increase asthma risk. Further, psychosocial stressors arising from the adaptation process in the new environment and family dissolutions consequent to migration could contribute to an increase in wheeze/asthma in migrant populations.^35–37^ A high proportion of migrants in LMICs are women who provide the primary economic support for their families working in urban areas in unskilled service jobs.^38^ In our study, 75% of the children with history of rural migration lived in families with one parent or without parents (table 2). As we have seen previously, the absence of parents at home (especially the mother) is an important determinant in the increase of wheeze in children of migrant parents.^13^ Another factor that could explain the higher prevalence of wheeze in rural to urban migrants could be migration in search of medical attention for asthma (reverse causality), emphasising the importance of the migration process in the spatial and temporal distribution of asthma between urban and rural areas.

The study of migration using various categories provides a better understanding of the possible factors and mechanisms affecting the development of asthma in urban areas of LMICs. For example, in addition to ‘direction of migration’ which classified migrants according to rural or urban birthplaces, we used ‘locality of migration’ to describe more precisely the possible social and physical environment of the previous residence of the children. In our study, for example, migrants from medium cities (capital cities of Ecuadorian provinces) had 68% more wheeze than non-migrants. We also identified an important migration flow from Colombia, especially from Nariño Department, a region that borders the province of Esmeraldas and which is located less than 150 km from the city of Esmeraldas (figure 1). Although this is a group of international migrants, they provided a useful comparison group because of proximity and a social and ecological environment similar to that found in Esmeraldas Province. Children born in Colombia or born of Colombian parents had a greater prevalence of wheeze compared with Ecuadorian children. Many of these migrants are involuntary migrants or refugees fleeing guerrilla and paramilitary violence in Colombia and the higher prevalence of wheeze in this population might be explained by psychosocial stressors related to displacement rather than changes in lifestyles.^39–41^ However, our study is subject to several limitations. First, the cross-sectional design does not permit assumptions of the direction of causality. Second, misreporting of birthplace related to recall bias and misclassification is possible, especially for boundary changes in the geographical units of study. However, the use of place of birth to define migration status is more precise than previous studies that have either not defined migration status or used other variables (eg, use of ethnic surnames as a surrogate marker for migrant status^15^). Further, specific information about the history of migration of the parents was limited. Third, not all wheezing is asthma, although this deﬁnition is probably more useful in rural populations where access to healthcare is limited and where alternative definitions such as doctor diagnosis may be subject to significant misclassification. Fourth, because selection of schools resulted in a sample of predominantly Afro-Ecuadorian schoolchildren (to represent the original source population of migrants from rural districts in the north of the province), our findings cannot necessarily be generalised to populations of differing ethnic compositions within the province. However, we believe that our findings provide novel insights into how social and demographic factors may affect asthma burden in LMICs.

Clearly, internal migration is a major contributor to the urbanisation process in LMICs and has a direct effect on the prevalence of asthma in urban and rural populations.^42^ It is possible that the differences in asthma prevalence between urban and rural areas or between LA countries could be in part explained by different rates of urbanisation and migration within the region. As for international migrants, internal migrants also face important economic, social and environmental changes, especially those associated with changes in diet, physical activity, housing, family composition and air pollution, all factors related to asthma risk.^43^ Special consideration must be given to rural migrants living in poor conditions in their new urban environments, generally slums and informal settlements. Populations living in such environments are exposed to a number of factors that could increase the risk of asthma or exacerbate existing disease such as inadequate housing, increased risk of respiratory infections through overcrowding and high levels of violence.^29^ Finally, migration results in exposure to a new set of pollutants and allergens, and to new socioeconomic and cultural factors resulting in important changes to the social and family environment in which the new arrivals find themselves and which may contribute to asthma risk. A better understanding of the effects of migration on asthma will allow us to identify potential public health interventions that could be tested with the aim of alleviating the growing burden of asthma disability, particularly among the urban poor of LA.

## Conclusion

We evaluated the effects of different migrant categories on the prevalence of wheeze in an urban area of LA. Our study provides evidence that rural to urban migration is a risk factor for wheezing in urban schoolchildren. Age of migration and time since migration were associated with an increased risk of wheeze only for rural to urban migrants but not for urban to urban migrants. Temporal, spatial and socioeconomic dimensions of the migration process may have different effects on the prevalence of wheeze/asthma and other allergic diseases. Further studies in different populations living in rural and urban areas of LMICs, that are subject to migration processes, are required in which detailed information is collected at individual, household and community levels.
